# Comparative Efficacy and Safety of Potassium-Competitive Acid Blocker– and Proton Pump Inhibitor–Based Bismuth Quadruple Therapy for *Helicobacter pylori* Eradication: A Network Meta-Analysis

**DOI:** 10.1016/j.gastha.2025.100705

**Published:** 2025-05-16

**Authors:** Iqbal Taufiqqurrachman, Ari Fahrial Syam, Hasan Maulahela, Murdani Abdullah, Muhammad Miftahussurur, Yoshio Yamaoka

**Affiliations:** 1 Department of Environmental and Preventive Medicine, Oita University Faculty of Medicine, Yufu, Japan; 2 Faculty of Medicine, Universitas Indonesia, Jakarta, Indonesia; 3 Division of Gastroenterology, Department of Internal Medicine, Faculty of Medicine, Universitas Indonesia, Cipto Mangunkusumo General Hospital, Jakarta, Indonesia; 4 Human Cancer Research Center, Indonesian Medical and Research Education Institute, Faculty of Medicine, Universitas Indonesia, Jakarta, Indonesia; 5 Division of Gastroentero-Hepatology, Department of Internal Medicine, Faculty of Medicine-Dr. Soetomo Teaching Hospital, Surabaya, Indonesia; 6 *Helicobacter Pylori* and Microbiota Study Group, Institute Tropical Disease, Universitas Airlangga, Surabaya, Indonesia; 7 Department of Medicine, Gastroenterology and Hepatology Section, Baylor College of Medicine, Houston, Texas; 8 Division of Genome-Wide Microbiology, Research Center for Global and Local Infectious Diseases (RCGLID), Oita University, Oita, Japan

**Keywords:** Bismuth quadruple therapy, Esomeprazole, *Helicobacter pylori*, Tegoprazan, Vonoprazan

## Abstract

**Background and Aims:**

The eradication of *Helicobacter pylori (H. pylori)* reduces the incidence of gastric cancer. However, the efficacy of the widely used triple therapy for eradicating *H. pylori* has progressively reduced. This may have resulted from the increase in clarithromycin resistance in recent years. Recent guidelines recommend the use of bismuth quadruple therapy (BQT) as first-line eradication therapy for *H. pylori* infection in areas with high (>15%) or unknown clarithromycin resistance. However, the eradication rates of proton pump inhibitor (PPI)-based BQT remain below the required standard. This systematic review aimed to evaluate the use of novel acid suppressant (potassium-competitive acid blocker [P-CAB])-based BQT compared with PPI-based BQT for *H. pylori* eradication.

**Methods:**

A systematic review and network meta-analysis were conducted using the PubMed, Cochrane Library, ProQuest, and Scopus databases, along with randomized controlled trials comparing P-CAB-based and PPI-based BQT for *H. pylori* eradication.

**Results:**

Intention-to-treat analysis showed a pooled risk ratio (RR) of 1.04 (95% confidence interval: 1.02–1.06, I^2^ = 0) and per-protocol set analysis yielded an RR of 1.04 (95% confidence interval: 1.01–1.07, I^2^ = 0), favoring P-CAB-based BQT with minimal heterogeneity. Seven studies (n = 2222) reported no significant difference in adverse events (RR: 1.06 [0.99–1.14, I^2^ = 30.6%]).

**Conclusion:**

Meta-analysis showed the P-CAB-based BQT had slightly higher efficacy than PPI-based BQT. However, network meta-analysis revealed that vonoprazan-based BQT did not show superiority over esomeprazole-based BQT. Therefore, tailored therapies based on local resistance patterns remain critical considerations in clinical practice.

## Graphical abstract

**Figure:**
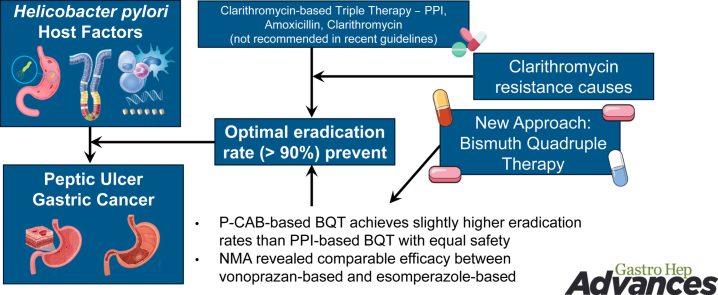

## Introduction

*Helicobacter pylori* (*H. pylori*) is a gram-negative, microaerophilic, helix-shaped bacterium expressing flagella that colonizes the gastric epithelium.^1^ It is a common cause of chronic gastritis and leads to peptic ulcer disease, duodenal ulcer, gastric cancer, and gastric mucosa-associated lymphoid tissue lymphoma.^2^^,^^3^ The crude global prevalence of *H. pylori* infection has decreased from 52.6% (before 1990) to 43.9% (between 2015 and 2022). In addition, the alteration of long-term prevalence pattern in the adult population have shown a 15.9% reduction in the global prevalence of *H. pylori* infection over the last 3 decades. In particular, significant decreases in prevalence have occurred in two specific geographical regions, the Western Pacific and Africa.^4^

The connection between *H. pylori* infection and the development of gastric cancer, along with host behavioral factors (such as smoking, alcohol consumption, high salt intake, and a low-fruit diet),^5^^,^^6^ likely contribute to the current high incidence of gastric cancer, with 1,000,000 new cases and 768,000 deaths in 2020, particularly affecting East Asia.^7^ However, this number has already declined in line with lower *H. pylori* infection rates.^8^ In patients, successful eradication of *H. pylori* infection lowers the gastric cancer incidence compared with a lack of eradication (risk ratio [RR] = 0.53).^9^ In addition, eradication therapy also reduces gastric inflammation and stimulates ulcer healing.^10^^,^^11^ Therefore, the “test-and-treat” approach was recommended for detecting and eradicating *H. pylori* infection in dyspeptic patients.^12^^,^^13^

Although eradication therapy is well established for *H. pylori*, challenges exist in its efficacy.^14^ The widely used *H. pylori* eradication regimen, known as triple therapy (proton pump inhibitor [PPI], amoxicillin, and clarithromycin), has shown a significant reduction in its eradication capability in recent times.^13^ This may be associated with the emergence of a high level of resistance to clarithromycin (35.3%).^15^ To maintain a minimal eradication rate of 90%,^13^ other approaches should be considered, including bismuth quadruple therapy (BQT). The use of BQT is currently recommended as the first-line eradication therapy for *H. pylori* infection in areas with high (>15%) or unknown clarithromycin resistance.^16^ A network meta-analysis (NMA) showed the superiority of the eradication rate of BQT (85%) to that of clarithromycin triple therapy (73%).^17^ However, the eradication rates of BQT, which range from 79.5%–86.6%, are still below the minimal target for the standard eradication rate (>90%).^18^^,^^19^ This shortcoming may be caused by gastric acid suppression stability with conventional BQT that uses a PPI, which is significantly affected by the host Cytochrome P450 2C19 (CYP2C19) genotype.^20^^,^^21^

To address this issue, a recently developed acid-suppression agent, known as a potassium-competitive acid blocker (P-CAB), was considered. This drug may be more stable than PPI, which is rapidly metabolized by the CYP2C19 early metabolizer.^22^ This systematic review aimed to evaluate the use of P-CAB-based BQT compared with PPI-based BQT for the eradication of *H. pylori* infection.

## Methods

### Search Strategy

The search was performed using the following four different databases: (1) PubMed, (2) The Cochrane Library, (3) ProQuest, and (4) Scopus. The literature search was conducted from December 30, 2024, to January 4, 2025. No restrictions were applied regarding the publication date of eligible studies. The search strategy was based on the population with *H. pylori* infection, intervention with P-CAB-based BQT, comparison with PPI-based BQT, and the outcomes of eradication rate (intention-to-treat [ITT], per-protocol set [PPS]), and adverse events.

### Eligibility Criteria

The inclusion criteria were as follows: (1) randomized controlled trial study from adult patients (>18 year); (2) patients diagnosed with *H. pylori* infection (based on a urea breath test, rapid urease test, culture, PCR, or a sequencing-based method); (3) grouped into: (a) P-CAB-based BQT and (b) PPI-based BQT as the first-line treatment of *H. pylori* infection; (4) no prior *H. pylori* eradication therapy; and (5) the outcomes of the study were eradication rate based on ITT and PPS, and adverse events. The exclusion criteria were as follows: (1) nonrandomized trials, and (2) single-arm studies or different comparisons.

### Data Collection, Selection, and Extraction

Data selection was performed independently by all reviewers, starting with the title and abstract, and the eligibility criteria were evaluated. After each reviewer created the database, the final selection of the article was analyzed based on discussion. If a conflict occurred during selection, a voting mechanism was employed. The extracted data included the study author and year of publication, country, subject characteristics, P-CAB use, PPI use, therapy duration, eradication rate (ITT, PPS), and adverse events.

### Statistical Analysis

The pooled effect size is represented as the RR of eradication rates (ITT and PPS) and adverse events. The pairwise meta-analysis was performed using the RR with a random-effects model in R studio (Version 2024.09.0 + 375, © Posit Software, PBC) using meta package.^23^ The pooled size effect was presented as a forest plot. Publication bias with funnel plots and Egger’s regression test were not performed because the total number of selected studies was less than 10 (k < 10).

To address the limitations of conventional pairwise meta-analysis, NMA was conducted to enable direct and indirect comparisons among the included regimens. A random-effects model was employed to account for between-study variability, and the surface under the cumulative ranking curves (SUCRA) probabilities were calculated to rank the treatments. The analysis was performed using the netmeta package in R, and the results were visualized using network and forest plots. By incorporating both direct and indirect evidence, NMA allowed for a more nuanced comparison of regimens, particularly between P-CAB-based and PPI-based BQT.^24^^,^^25^

### Risk of Bias Assessment

The risk of bias was assessed using the Revised Cochrane Risk-of-Bias Tool for Randomized Trials by Cochrane.^26^ This assessment tool comprised the following five domains: (1) bias arising from the randomization process; (2) bias due to deviations from intended interventions; (3) bias due to missing outcome data; (4) bias in measurement of the outcome; and (5) bias in selection of the reported result.^26^

## Results

### Study Selection

The search was performed using four different databases: PubMed, Cochrane Library, ProQuest, and Scopus, from December 30, 2024, to January 4, 2024. There were 834 search results, with 33 articles that matched the titles and abstracts. After the eligibility criteria were evaluated, 12 eligible articles were included. After deduplication, eight articles were included in the meta-analysis (Figure A1).

### Risk of Bias in Studies

Based on a risk of bias of 2.0, most included studies demonstrated a low risk of bias across all domains, reflecting strong methodological quality and reliability. The studies by Wang J et al. and Zhou et al. were not included in the formal risk of bias assessment because only abstracts were available at the time of analysis, making it difficult to perform a comprehensive evaluation of their methodological quality and potential sources of bias. Overall, the cumulative assessment confirmed that most studies included in the systematic review and meta-analysis had a low risk of bias, supporting the robustness and validity of the evidence presented (Figure A2).

### Study Characteristics

The study characteristics are summarized in Table 1.

**Table 1:** Study Characteristics

Study author	Year	Country	Type of study	Number of patients	Methods	*H. pylori* diagnostic modalities and posttreatment evaluation		P-CAB-based BQT protocol		PPI-based BQT Protocol	
						Diagnosis	Evaluation posttreatment	Protocol	Frequency and duration	Protocol	Frequency and duration
Wang J, et al.^27^	2020	China	Double-blind RCT, phase III, multicenter	433	**Eligibility criteria** - Adult Chinese patient - Diagnosis of duodenal ulcer confirmed by endoscopy - *H. pylori* infection confirmed (specfici diagnostic modality not mentioned) **Study protocol** Eligible participants were assigned to one of two groups: (1) Vonoprazan-based BQT; or (2) lansoprazole-based BQT The treatment was administered for 14 d. Follow-up was conducted using the^13^C-urea breath test (UBT).	Not mentioned	^13^C-UBT	Vonoprazan-based BQT (vonoprazan 20 mg combined with BQT but the specific components no detailed)	The regimen was administered twice daily for 14 d	Lansoprazole-based BQT (lansoprazole 30 mg combined with BQT but the specific components not detailed)	The regimen was administered twice daily for 14 d
Kim JS, et al.^28^	2023	South Korea	Double blind RCT, noninferiority study	217	**Eligibility criteria** - Adult patients aged 19–75 y - Outpatiens who underwent upper GI endoscopy - Confirmed *H. pylori* infection based on rapid urease test (RUT) or histological examination **Exclusion criteria:** (1) prior *H .pylori* eradication therapy; (2) history of gastric cancer surgery; (3) history of malignancy (other than gastric cancer) within the last 5 y, clinically significant hepatic or renal disease, hematologic or neurologic disorders or infectious mononucleosis **Study protocol** Eligibility screening was conducted through physical examination, laboratory tests, pregnancy testing, and ECG, followed by upper GI endoscopy. Diagnosis of *H. pylori* infection was confirmed via RUT or histological examination. Antimicrobial susceptibility testing (AST) was performed using gastric mucosal tissue. Eligible patients were then randomly assigned to either the tegoprazan-based BQT group or the lansoprazole-based BQT group. Follow-up was conducted using the urea breath test (UBT) 28 d (4 wk) after completion of therapy. At the end of the treatment period, physical and laboratory examinations were performed to assess adverse events, concomitant medication use, and treatment compliance.	RUT or histologic examination	UBT	TBMT (tegoprazan 50 mg and placebo twice daily; metronidazole 500 mg three times a day; tetracycline 500 g four times a day; and bismuth subcitrate (DE-Nol) 300 mg four times a day)	The regimen was administered by different frequency for specific components for 14 d	LBMT (lansoprazole 30 mg and placebo twice daily; metronidazole 500 mg three times a day; tetracycline 500 g four times a day; and bismuth subcitrate (DE-Nol) 300 mg four times a day)	The regimen was administered by different frequency for specific components for 14 d
Lu L, et al.^29^	2023	China	RCT, noninferiority study, single centre	234	**Eligibility criteria** - Adult patients aged 18–65 y - Endoscopy was performed in subjects with alarm symptoms, age >35 y, or a family history of gastric cancer - Diagnosis of *H. pylori* was based on gastric biopsy (histochemical staining and/or tissue culture),^14^C-UBT, and/or^13^C-UBT **Exclusion criteria:** (1) use of antibiotics or bismuth within 4 wk before the study (2) Use of acid-suppressing therapy (H_2_-receptor antagonists, PPIs, or P-CABs) within 2 wk before the study (3) Active peptic ulcer with complications (hemorrhage, perforation, or obstruction) (4) History of gastrectomy or esophagectomy (5) Known allergy to any study drug (6) Severe comorbidities or physical/mental illness (7) Pregnant or breastfeeding women (8) History of alcohol abuse or drug addiction **Study protocol** Eligible participants were assigned to one of three groups: (1) 10-d vonoprazan-based BQT (2) 14-d vonoprazan-based BQT (3) 14-d esomeprazole-based BQT The study was open-label; both participants and researchers were aware of the assigned treatments. However, personnel performing the UBT were blinded to treatment allocation. *H. pylori* eradication was assessed using the^13^C-UBT performed 6–8 wk after the completion of therapy.	Histology examination, tissue culture,^14^C-UBT, and/or 13C-UBT	^13^C-UBT	Vonoprazan-quadruple (vonoprazan 20 mg once daily, combined with amoxicillin 1000 mg, furazolidone, and bismuth potassium citrate 240 mg, all administered twice daily.) Colloidal bismuth was given 30 min before breakfast and dinner, while amoxicillin and furazolidone were administered immediately after breakfast and dinner.	The frequency of administration varied for each drug. The duration of treatment was 10 d for group 1 and 14 d for group 2	Esomperazole-quadruple (esomeprazole 20 mg once daily, along with amoxicillin 1000 mg, furazolidone, and bismuth potassium citrate 240 mg, all administered twice daily. Both colloidal bismuth and esomeprazole were taken 30 min before breakfast and dinner, whereas amoxicillin and furazolidone were taken immediately afterward)	The frequency of administration differed by drug. The total duration of therapy in this group was 14 d
Song Z, et al.^30^	2023	China	Double blind RCT, phase III, multicenter	510	**Eligibility criteria** Eligible participants were adult patients aged 18 y or older with confirmed *Helicobacter pylori* infection. The evaluation of eradication was based on the results of the^13^C-urea breath test (^13^C-UBT). **Study protocol** Participants were randomly assigned to one of the following three groups: (1) 14-d vonoprazan-based BQT, and (3) 14-d esomeprazole-based BQT. The efficacy of *H. pylori* eradication was assessed at 4 wk after the completion of therapy.	Not mentioned	^13^C-UBT	Vonoprazan-quadruple (vonoprazan 20 mg; amoxicillin 1000 mg; clarithromycin 500 mg, and bismuth potassium citrate 600 mg)	Twice daily for 14 d	Esomeprazole-quadruple (esomeprazole 20 mg; amoxicillin 1000 mg; clarithromycin 500 mg, and bismuth potassium citrate 600 mg)	Twice daily for 14 d
Tan N, et al.^31^	2024	China	Double blind RCT, phase III, multicenter (30 hospitals)	573	**Eligibility criteria** Adult patients aged 18–70 y with confirmed *Helicobacter pylori* infection based on the^13^C-urea breath test (^13^C-UBT) were included. Patients were excluded if they had any of the following: a History of *H. pylori* eradication therapy; allergy to any study drug; history of gastrointestinal disease or any condition that could influence study outcomes (eg, upper GI bleeding, acute gastric or duodenal mucosal lesions, active gastric or duodenal ulcer, malignancy, or Zollinger–Ellison syndrome); serious systemic illness; history of gastric surgery affecting acid secretion; renal or hepatic dysfunction; use of PPIs or P-CABs within 2 wk prior to the^13^C-UBT; prior use of antibiotics or bismuth-containing formulations within 4 wk prior to the^13^C-UBT; a history of drug or alcohol abuse. **Study protocol** The study consisted of three phases: (1) a 1-wk screening phase, (2) a 2-wk treatment phase, and (3) a 4-wk follow-up phase. Eligible patients were randomized to receive either keverprazan-based BQT or esomeprazole-based BQT orally. Several additional assessments were performed, including: (1) endoscopy to exclude patients meeting exclusion criteria, (2) CYP2C19 genotyping (3) Clarithromycin resistance testing, and (4) physical examination and electrocardiogram (ECG) during the screening period and at week 2. Follow-up assessment was performed 4 wk after the completion of eradication therapy. Testing for *H. pylori* was conducted at both the screening phase and at week 6.	^13^C-UBT	^13^C-UBT	Keverprazan-quadruple (keverprazan 20 mg; Claritrhomycin 500 mg; amoxicillin 1000 mg; bismuth potassium citrate 240 mg) The therapy was administered before breakfast and dinner, while the antibiotics were taken after meals.	Twice daily for 14 d	Esomeprazole-quadruple (esomeprazole 20 mg; Claritrhomycin 500 mg; amoxicillin 1000 mg; bismuth potassium citrate 240 mg) The therapy was administered before breakfast and dinner, while the antibiotics were taken after meals.	Twice daily for 14 d
Chen C, et al.^32^	2024	China	RCT, noninferiority study, single centre	135	Adult patients aged 18–75 y with confirmed *Helicobacter pylori* infection based on the^13^C-urea breath test (^13^C-UBT) were included. Patients had no history of severe liver insufficiency, renal insufficiency, or gastrointestinal tumors, and had not used proton pump inhibitors (PPIs), antibiotics, or other related medications within the past 4 wk. **Exclusion criteria included:** (1) history of gastrointestinal tumors or surgery; (2) Known allergy to any of the study drugs; (3) Presence of severe mental disorders that impaired communication; (4) Refusal to participate in the study. **Study protocol** Eligible participants were randomized into three treatment groups: (1) Vonoprazan 20 mg and amoxicillin 1 g, administered three times daily for 14 d; (2) Vonoprazan 20 mg, amoxicillin 1 g, furazolidone 0.1 g, and bismuth potassium citrate 240 mg; (3) Ilaprazole 20 mg, amoxicillin 1 g, furazolidone 0.1 g, and bismuth potassium citrate 240 mg. All treatment regimens were administered for 14 d. One month (4 wk) after completing therapy, *H. pylori* eradication was evaluated using the^13^C-UBT.	^13^C-UBT	^13^C-UBT	Vonoprazan-quadruple (vonoprazan 20 mg; and furazolidone 0.1 gr; amoxicillin 1000 mg; bismuth potassium citrate 240 mg) Vonoprazan and bismuth were administered 30 min before meals, while antibiotics were given 30 min after meals.	Twice daily for 14 d	Ilaprazole-quadruple (ilaprazole 20 mg; and furazolidone 0.1 gr; amoxicillin 1000 mg; bismuth potassium citrate 240 mg) Ilaprazole and bismuth were administered 30 min before meals, while antibiotics were given 30 min after meals.	Twice daily for 14 d
Zhou L, et al.^33^	2024	China	Double-blind RCT, phase III, multicenter	555	**Eligibility criteria** Adult Chinese patients with confirmed *Helicobacter pylori* infection (diagnostic method not specified) were enrolled. Eligible patients were assigned to either a tegoprazan-based or esomeprazole-based bismuth quadruple therapy (BQT) group. *H. pylori* eradication was evaluated using the^13^C-urea breath test (^13^C-UBT) after treatment. **Study protocol** Participants were randomized into two treatment groups: (1) tegoprazan-based BQT and (2) esomeprazole-based BQT. The treatment duration was 14 d. Posttreatment eradication was assessed using the^13^C-UBT, although the specific timing of the test was not reported.	Not mentioned	^13^C-UBT	Tegoprazan-based BQT (tegoprazan 20 mg and bismuth quadruple therapy) however, the detailed information was not reported	The frequency of administration was not specified. The duration of therapy was 14 d.	Esomeprazole-based BQT (esomeprazole 20 mg and bismuth quadruple therapy) however, the detailed information was not reported	The frequency of administration was not specified. The duration of therapy was 14 d.
Hou X, et al.^34^	2024	China Taiwan South Korea	Pooled two phase III, multicenter, randomized, double-blind RCT, double-dummy, parallel-group, noninferiority	629	**Eligibility criteria** Adult patients aged 18 y or older with a confirmed *Helicobacter pylori* infection based on the^13^C-urea breath test (^13^C-UBT) were included. Patients had no history of *H. pylori* eradication therapy outside of the study protocol. Eligible participants had endoscopic evidence of active peptic ulcers—either duodenal ulcers (DU) or gastric ulcers (GU)—with a minimum size requirement, identified within 14 d prior to randomization. **Exclusion criteria:** • Presence of linear ulcers, active postoperative ulcers, or acute peptic mucosal lesions within 14 d before randomization • History or recent treatment (within 5 y) for malignancy • Diagnosis of Zollinger–Ellison syndrome or gastric acid hypersecretion • Hepatitis • Use of medications that could interfere with UBT results • Prior use (within 30 d before treatment) of antibiotics, antiprotozoals, medications contraindicated with clarithromycin, or strong CYP3A4 inducers/inhibitors • Known hypersensitivity to vonoprazan or lansoprazole • Serum creatinine level > 2 mg/dL • ALT, AST, or total bilirubin levels above the upper limit of normal prior to randomization **Study protocol** Participants were randomly assigned to one of two treatment groups: (1) vonoprazan-based BQT, or (2) lansoprazole-based BQT. The study consisted of three phases: (1) a screening period (up to 28 d), (2) A treatment period (6–8 wk), and (3) a follow-up period (up to 4 wk). *H. pylori* eradication was evaluated using the^13^C-UBT, performed 4 wk after completion of eradication therapy.	^13^C-UBT	^13^C-UBT	Vonoprazan-based BQT (vonoprazan 20 mg, amoxicillin 1 gr, clarithromycin 500 mg, and bismuth potassium citrate/bismuth tripotassium dicitrate 600 mg [equivalent to bismuth 220 mg])	Twice daily for 14 d	Lansoprazole-based BQT (lansoprazole 30 mg, amoxicillin 1 gr, clarithromycin 500 mg, and bismuth potassium citrate/bismuth tripotassium dicitrate 600 mg [equivalent to bismuth 220 mg])	Twice daily for 14 d

AST: antibiotic/antimicrobial sensitivity test; BQT: bismuth quadruple therapy; DU: duodenal ulcer; ECG: electrocardiogram; GI: gastrointestinal; GU: gastric ulcer; LBMT: lansoprazole-based BQT; RUT: rapid urease test; TBMT: tegoprazan-based BQT; UBT: urea breath test.

### Data Synthesis

#### Efficacy of P-CAB-based BQT versus PPI-based BQT as first-line *H. pylori* eradication treatment

**Figure 1:**
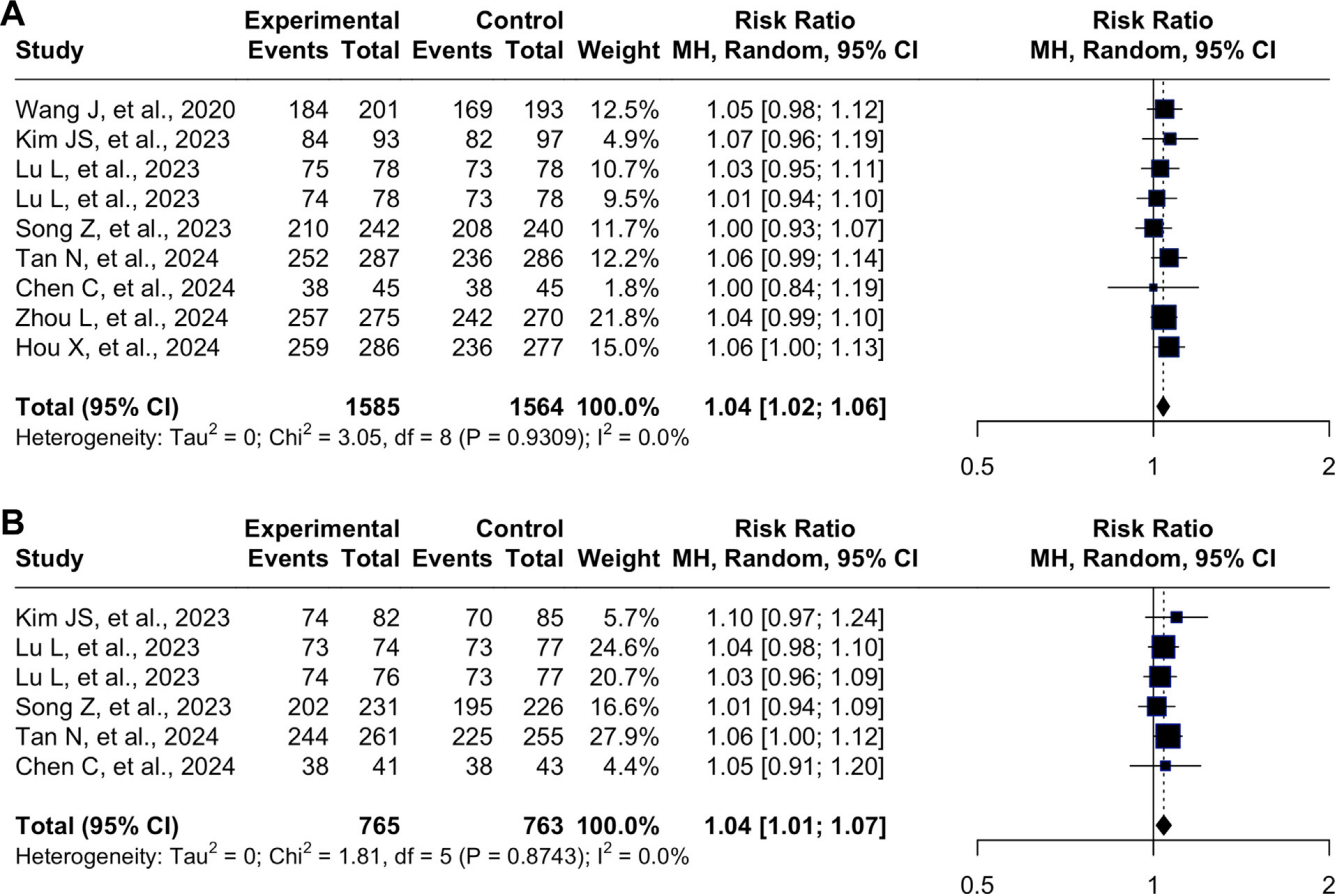
Individual Forest Plot of ITT and PPS of P-CAB-based (Experimental) vs PPI-based BQT (Control) as the First Line *H. pylori* Eradication Therapy. (A) The Forest Plot of ITT Eradication Rate P-CAB vs PPI-based BQT as First Line of *H. pylori* Eradication Regimens. (B) The Forest Plot of PPS Eradication Rate P-CAB vs PPI-based BQT as First Line of *H. pylori* Eradication Regimens. Experimental = P-CAB-based BQT; Control = PPI-based BQT.

Table 2 summarizes the efficacy and safety of these treatments. Figure 1. show the pooled analyses of studies evaluating the RR of *H. pylori* eradication between P-CAB-based and PPI-based therapies as first-line eradication treatments, including both ITT and PPS analyses. In the ITT analysis, which included a total of 3149 participants from seven studies, the pooled RR was 1.04 (95% confidence interval [CI]: 1.02–1.06, I^2^ = 0), favoring P-CAB-based therapy over PPI-based therapy. The heterogeneity for ITT was low and the Chi^2^ test was nonsignificant (*P* = .9309), indicating consistency across studies. For the PPS analysis, which included a total of 1457 participants from six studies, the pooled RR was 1.04 (95% CI: 1.01–1.07, I^2^ = 0), also favoring P-CAB-based therapy. Similarly, heterogeneity was minimal and the Chi^2^ test was nonsignificant (*P* = .874). The combined dataset reinforces that P-CAB-based therapy has a slightly higher efficacy than PPI-based therapy for *H. pylori* eradication, with minimal heterogeneity observed across all analyses (I^2^ = 0%).

**Table 2:** Efficacy and Safety Profile of P-CAB-Based vs PPI-Based BQT as the First Line *H. pylori* Eradication Treatment

Study author	Year	Number of patients	Efficacy (intention-to-treat and per-protocol set)						Treatment differences		Safety	
			ITT^a^ P-CAB-based	ITT PPI-based	*P* Value ITT	PPS^b^ P-CAB-based	PPS PPI-based	*P* Value PP	ITT	PPS	Any side effect of PCAB-based	Any side effect of PPI-based
Wang J, et al.^27^	2020	394	184/201 (91.50%)	169/193 (87.60%)	< .001	N/A	N/A	N/A	4 (−2.06-10.07)	N/A	N/A	N/A
Kim JS, et al.^28^	2023	217	84/93 (90.30%)	82/98 (84.50%)	.0005	74/82 (90.20%)	70/85 (82.40%)	.0003	5.80 (−3.6-15.2)	7.9 (−2.5-18.2)	41/105 (39.10%)	46/106 (43.40%)
Lu L, et al.^29^	2023	156	75/78 (96.20%)	73/78 (93.6%)	.002	73/74 (98.60%)	73/77 (94.80%)	< .001	2.56 (−6.08-11.2)	3.84 (−4.54-12.23)	10/78 (12.80%)	5/78 (6.40%)
Lu L, et al.^29^	2023	156	74/78 (94.90%)	73/78 (93.60%)	.006	74/76 (97.40%)	73/77 (94.80%)	.002	1.28 (−7.46-10.02)	2.56 (−5.79-10.92)	3/78 (3.80%)	5/78 (6.40%)
Song Z, et al.^30^	2023	510	210/242 (86.80%)	208/240 (86.70%)	.0009	202/231 (87.40%)	195/226 (86.30%)	.0002	0.10 (−5.95-6.17)	1.20 (−5.03-7.36)	167/242 (69.00%)	147/240 (61.25%)
Tan N, et al.^31^	2024	573	252/287 (87.80%)	236/286 (82.52%)	.075	244/261 (93.49%)	225/255 (88.24%)	.0382	5.29 (−0.55-11.18)	5.25 (0.29–10.45)	219/287 (76.31%)	222/286 (77.62%)
Chen C, et al.^32^	2024	135	38/45 (84.40%)	38/45 (84.40%)	.095	38/41 (92.70%)	38/43 (88.40%)	.186	N/A	N/A	8/41 (19.50%)	6/41 (14.00%)
Zhou L, et al.^33^	2024	555	257/275 (93.35%)	242/280 (86.40%)	.0055	N/A	N/A	N/A	7 (2.06–11.99)	N/A	210/278 (75.50%)	207/282 (73/4%)
Hou X, et al.^34^	2024	629	259/286 (90.60%)	236/277 (85.20%)	< .001	N/A	N/A	N/A	5.4 (−0.1-10.8)	N/A	226/311 (72.7%)	199/318 (62.60%)

a ITT: intention to treat (the analysis result of eradication rate from all patients based on randomization group after the therapy was given).

b PPS: per protocol set (the analysis of result of eradication rate from only the patients who finished the protocol completely and correctly); the *P* values presented in this table refer to the results of noninferiority test as reported or calculated based on the original study designs.

Pairwise meta-analysis can only evaluate either P-CAB or PPI-based BQT but cannot determine which protocol has the best effect. To address these limitations and evaluate the comparative efficacy of specific protocols, a NMA was performed, which included nine studies and evaluated six distinct regimens: (1) P-CAB-based, vonoprazan, tegoprazan, and keverprazan, and (2) PPI-based, esomeprazole, lansoprazole, and ilaprazole. The network was well connected, with direct and indirect comparisons between regimens (Figure A3). Esomeprazole-based therapy was chosen as the reference treatment because of its widespread use and clinical effectiveness, which provided a stable baseline for comparison.

**Figure 2:**
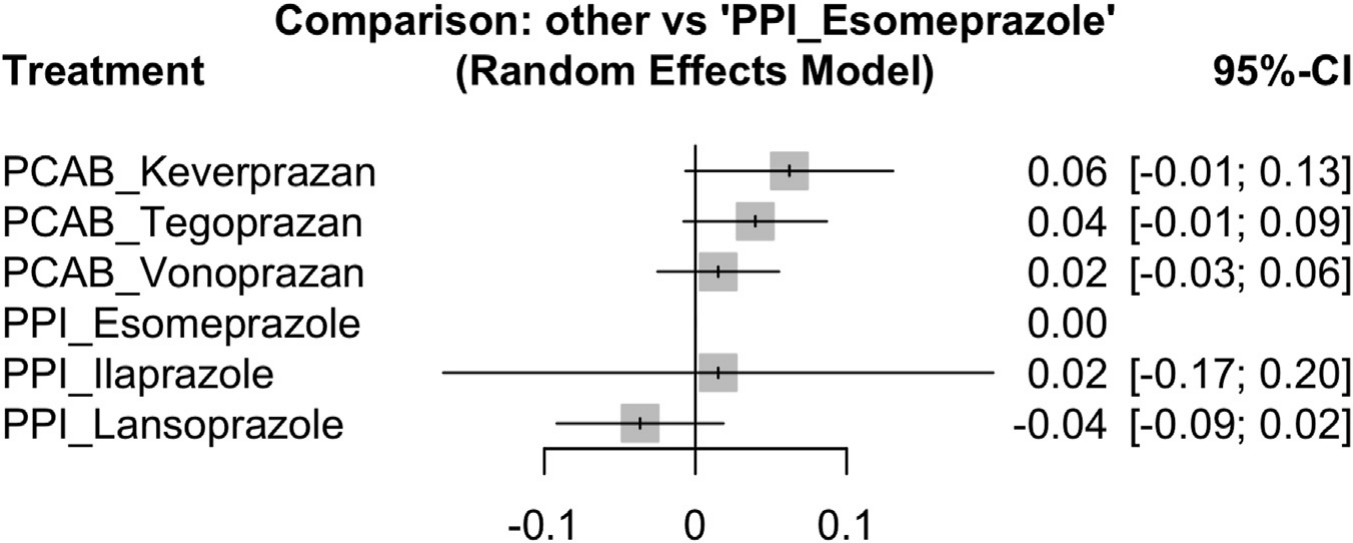
Forest plot of pairwise comparison of network meta analysis.

In the random-effects model (Figure 2), the keverprazan-based treatment showed a trend toward higher eradication rates than the esomeprazole-based treatment (treatment effect (TE) = 0.06; 95% CI: −0.01–0.13; *P* = .0759), although the difference was not statistically significant. Similarly, tegoprazan-based (TE = 0.04; 95% CI: −0.01–0.09; *P* = .1019) and vonoprazan-based (TE = 0.02; 95% CI: −0.03–0.06; *P* = .4614) methods were not significantly different from esomeprazole-based methods. In addition, both ilaprazole-based (TE = 0.02; 95% CI: −0.17–0.20; *P* = .8706) and lansoprazole-based (TE =−0.04; 95% CI: −0.09–0.02; *P* = .1937) methods showed no statistically significant differences from the reference.

The SUCRA probabilities provide additional insight into the relative rankings of the regimens (Table 3). The lansoprazole-based methods had the highest probability of ranking first (65.9%) despite not showing significant differences in pairwise comparisons. Vonoprazan-based BQT consistently ranked in the middle positions (3rd and 4th), reflecting stable but not superior performance. Esomeprazole-based data demonstrated a balanced distribution across ranks, confirming its role as a strong baseline comparator. Meanwhile, the keverprazan-based method ranked higher than the ilaprazole-based method, which was frequently positioned in the lower ranks. Heterogeneity across studies was minimal, with an I^2^ value of 0%, indicating high consistency in the network. The test of inconsistency revealed no significant differences between the direct and indirect evidence, strengthening the reliability of the results.

**Table 3:** SUCRA Rankings

Protocol	Rank 1 (%)	Rank 2 (%)	Rank 3 (%)	Rank 4 (%)	Rank 5 (%)	Rank 7 (%)
Keveprazan-based	0.6	3.5	5.4	11.5	28.4	50.6
Tegoprazan-based	0.6	2.9	9.0	25.9	43.6	18.0
Vonoprazan-based	0.1	9.1	36.1	40.8	13.1	0.8
Esomeprazole-based	4.4	41.6	38.6	12.4	3.0	0.0
Ilaprazole-based	28.4	13.6	7.1	8.7	11.6	30.6
Lansoprazole-based	65.9	29.3	3.8	0.7	0.3	0.0

### Safety of P-CAB-Based BQT versus PPI-Based BQT

**Figure 3:**
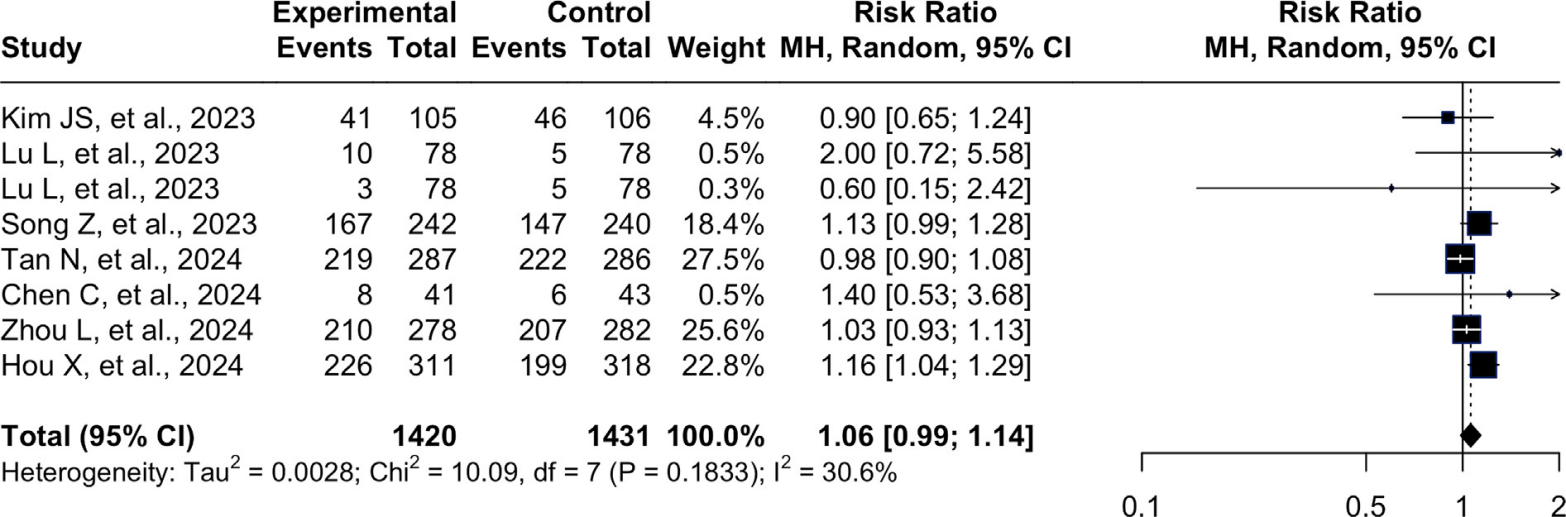
Forest Plot of Comparison of Safety between P-CAB-based vs PPI-based BQT as the First Line *H. pylori* Eradication Therapy. Experimental = P-CAB-based BQT; Control = PPI-based BQT.

The forest plots are shown in Figure 3 and represent pooled analysis of adverse events (safety outcomes) comparing P-CAB-based and PPI-based therapies across seven studies. There were 1420 participants in the experimental group (P-CAB-based BQT) and 1431 participants in the control group. A control group (PPI-based BQT) was included in this study. The overall pooled RR was 1.06 (0.99–1.14, I^2^ = 30.6%), indicating no significant difference in the risk of adverse events between P-CAB-based and PPI-based therapies. The individual study estimates showed some variation; however, the overall results supported the conclusion that P-CAB-based therapy is as safe as PPI-based therapy for adverse events.

## Discussion

Increasing clarithromycin resistance has reduced the efficacy of *H. pylori* eradication when triple therapy is prescribed, and BQT has been introduced to address this problem. A systematic review by Salahi-Niri *et al.* (2024) showed that the primary resistance rates of antibiotics were high, particularly for metronidazole (35.3%) and clarithromycin (32.6%).^15^ Along with the progression of clarithromycin resistance, which has increased steadily in recent years, the failure rate of triple therapy has risen to more than 20%.^35^ Triple therapy has therefore been replaced by quadruple therapy as the standard first-line treatment in most settings.^16^^,^^34^

The included studies demonstrated notable differences in intervention and comparison protocols for *H. pylori* eradication. Among P-CAB-based BQT, vonoprazan was the most commonly used P-CAB (5 studies), followed by tegoprazan (2 studies) and keverprazan (1 study). These were compared with PPI-based BQT, such as lansoprazole (3 studies), esomeprazole (4 studies), and ilaprazole (1 study). Most protocols include amoxicillin and clarithromycin as the core antibiotics, with additional variations, such as tetracycline and metronidazole in tegoprazan- and vonoprazan-based BQT regimens, or furazolidone in vonoprazan- and ilaprazole-based regimens. The included studies were conducted in Asian countries, and the trend toward BQT prescriptions has since progressed in Europe, particularly after the use of the single-capsule scheme. The proportion of BQT among all eradication therapies has increased from 8.6% in 2013 to 39% in 2021. Single-capsule BQT, which combines PPI, bismuth, metronidazole, and tetracycline into a single capsule, accounts for 43% of all bismuth-based treatments and is associated with an eradication rate of >90%.^27^

The duration of therapy was predominantly 14 days, except for one study assessing a 10-day protocol for vonoprazan. Of the included studies, the protocols in Hou X, *et al.*^28^ (2014) and Wang J, *et al.*^36^ (2020) shared particular similarity, as both compared vonoprazan-based BQT (including amoxicillin, clarithromycin, and bismuth) with lansoprazole-based BQT using the same antibiotics over 14 days. Similarly, Kim *et al.*^37^ (2023) and Zhou *et al.*^19^ (2024) administered tegoprazan- and esomeprazole-based BQT with identical antibiotic compositions (metronidazole, tetracycline, and bismuth) for 14 days. The 14-day regimens achieved superior eradication rates (RR: 1.04; 95% CI: 1.02–1.06; I^2^ = 0%) to the 10-day regimens. However, a report by Yang E, *et al.* (2024) showed noninferiority of 10-day BQT regimens (esomeprazole 40 mg twice daily, colloidal bismuth subcitrate 120 mg q.i.d., metronidazole 500 mg t.i.d., and tetracycline 500 mg q.i.d. for 10 days–14 days (92.4% vs 92.9%).^38^ This difference may reflect the limited data that used a 10-day protocol in our report.

The choice of antibiotics also varied across the studies, with amoxicillin and clarithromycin being the most commonly used core antibiotics in both P-CAB-based and PPI-based BQT protocols. Studies involving vonoprazan and tegoprazan frequently incorporated tetracycline and metronidazole, whereas furazolidone was uniquely included in the regimens with vonoprazan and ilaprazole. In a nonrandomized controlled trial, He *et al.* (2024), used minocycline and vonoprazan as BQT for *H. pylori* first-line treatment.^39^

In pairwise meta-analysis, P-CAB-based BQT was superior to PPI-based BQT as the first-line treatment for *H. pylori* infection, with consistently higher eradication rates observed across studies, even though the particular antibiotics varied. This result was also supported by He *et al.* (2024), who reported a high eradication rate (90.1%) with the use of vonoprazan, amoxicillin, minocycline, and colloidal bismuth pectin as the first-line treatment for *H. pylori*.^39^ The The NMA provided a detailed comparison to evaluate different regimens. P-CAB regimens, such as vonoprazan, tegoprazan, and keverprazan, showed a trend towards higher efficacy than PPI-based BQT, although these differences were not statistically significant compared to esomeprazole, the chosen reference. The SUCRA rankings also highlighted lansoprazole and vonoprazan as consistently higher, suggesting their competitive efficacy. These results indicate that, while P-CAB regimens may have slightly higher eradication rates, certain PPI regimens such as esomeprazole remain robust and clinically relevant options.

These findings have reversed the status of pairwise meta-analysis favoring the use of P-CAB-based quadruple therapy for the first line regimen. A deeper understanding of the role of acid suppression is essential in this context. Maintaining the optimal intragastric pH is critical for antibiotic stabilization and improved *H. pylori* eradication.^40^ The P-CAB-based regimens have been shown to produce higher pH 4 holding time ratios (HTRs). A report by Ke H, *et al.* (2020) reported that patients with successful eradication had a 24-hour media intragastric pH of 6.10 compared to 5.65 in those who failed treatment (*P* = .038). The eradicated group also had the higher pH 4 HTR (96%) than the failed group (87.5%).^41^ Similarly, a small study in Japan by Sakurai et al. (2015) was reported the significantly higher pH 4 HTR at day 1 and day 7 for vonoprazan than for esomeprazole and rabeprazole.^42^ A recent report summarized the studies showing the higher pH 4 HTR for vonoprazan 20 mg (88.2%) if compared to tegoprazan 100 mg (70.4%), esomeprazole 20 mg b.i.d. (73.9%), lansoprazole 30 mg (55.6%), omeprazole 40 mg (61.4%), pantoprazole 40 mg b.i.d. (70.8%), rabeprazole 10 mg b.i.d. (73.5%), and ilaprazole 10 mg (77.2%).^43^

In addition, the pharmacokinetic variability of PPIs, particularly their metabolism via CYP2C19 can affect the eradication outcomes. Rapid metabolizers tend to have lower intragastric pH levels when taking PPIs, reducing efficacy.^22^ This genetic polymorphism is known to significantly influence the efficacy of omeprazole and lansoprazole.^44^ These two mechanisms (higher HTR pH and unaffected by CYP2C19 genotype) favoured P-CAB over PPI in a pairwise meta-analysis.

However, if we evaluated to specific comparison between the specific acid suppressants, the esomeprazole is comparable to vonoprazan. The basic mechanism that could explain the resilience of esomeprazole compared to P-CAB is its pharmacodynamic and pharmacokinetic profile. First, a larger study bby Ke H, *et al.* (2020) showed a higher pH 4 and pH 5 HTR than another study for esomeprazole 20 mg with 91.1% and 83.3%.^41^ Second, although esomeprazole is affected by CYP2C19, when compared to other PPIs, such as omeprazole, the enzyme affected only 70% of its clearance rather than 90% for omeprazole. In addition, although clearance was reduced, the increase in the area under the curve with repeated administration of esomeprazole was greater than that with omeprazole.^45^

However, this finding does not fully explain why lansoprazole-based therapy outperformed vonoprazan-based therapy compared to esomeprazole-based therapy in the SUCRA analysis. It is therefore important to consider other unmeasured factors in this analysis, such as antibiotic susceptibility (particularly clarithromycin resistance in East Asian studies), bacterial genotypes, and patient-related factors such as smoking or treatment adherence, which may contribute to the observed variability.^40^^,^^41^

None of the studies mentioned long-term outcomes (recurrence rates and resistance patterns), which may be due to the novelty of the drugs. This is also a concern discussed in a recent meta-analysis of BQT by Gohar *et al.* (2024).^46^ A meta-analysis by Zhao *et al.* (2021) reported that the pooled recurrence rate of *H. pylori* infection was 9%, with an increasing trend after the first (4%), second (6%), third (8%), and fourth years or longer (12%). The recurrence rate of *H. pylori* infection in patients who received triple therapy was higher than in those who received quadruple therapy (14% vs 12%). Unfortunately, the subgroup analysis of recurrence rate per year after eradication for different types of eradication regimens or the details of regimens were not available.^47^ Studies that directly evaluated the long-term effects of BQT to resistance patterns were limited. However, the compliance rate of quadruple therapy could be a parameter for estimating the risk of long-term effects associated with resistance patterns, because nonadherence to antibiotics can lead to the development of antibiotic resistance.^48^ A report by Huguet *et al.* (2024) showed that the high noncompliance rates in the first-line treatment of *H. pylori* were from doxycycline-metronidazole BQT (6.7%) and amoxicillin-levofloxacin BQT (4.6%). Overall, the noncompliance group had a first-line eradication rate as low as 58%.^49^ This lack of compliance (possibly caused by the high pill burden, complex treatment course, or side effects of the regimens)^31^^,^^50^ could present a further potential risk of resistance.^29^^,^^30^

Instead of focusing only on acid suppressant drugs, the optimal efficacy of BQT may also be influenced by the resistance pattern of the regional study area, which was dominated by Asian countries (China, South Korea, and Taiwan). Lee J, *et al.* (2019) reported high resistance rates for clarithromycin (17.8%), metronidazole (29.5%), levofloxacin (37.0%), and ciprofloxacin (37.0%), but low resistance rates for amoxicillin (9.5%) and tetracycline (0.0%). A study in South Korea by Kim *et al.* (2023) used metronidazole-containing BQT, and their population showed high metronidazole resistance (46.4% in tegoprazan-based BQT and 60.0% in lansoprazole-based BQT). Even though the overall eradication rate was 90.2% in tegoprazan-based BQT, the eradication rates were low in the tegoprazan and lansoprazole groups with metronidazole resistance (76.9% vs 73.3%) and susceptibility (80.0% vs 60.0%). A recent meta-analysis by Chen *et al.* (2022) showed high resistance to clarithromycin (34%), metronidazole (78%), and levofloxacin (35%), with regional differences in clarithromycin resistance, which were higher in northern China (37%), followed by western China (35%), and were lower in southern and eastern China (24%).^32^ However, even though the clarithromycin resistance rate was high, clarithromycin-containing BQT still showed an acceptable eradication rate (86.80%–93.35%).^19^^,^^33^ However, local resistance patterns should be taken into account before these findings are applied clinically. For example, the possibility of implementing amoxicillin-containing regimens may be unacceptable in African countries that have an amoxicillin resistance rate as high as 72.6%.^51^

The safety profiles of the P-CAB-based and PPI-based BQT regimens were comparable, with both regimens showing similar rates of adverse events and treatment discontinuation. Common side effects included gastrointestinal disturbances, taste alterations, and headaches, which were classified as mild-to-moderate. Forest plot analysis indicated that P-CAB regimens, particularly vonoprazan, had a slightly lower, though not statistically significant, incidence of adverse events than PPI-based regimens, such as esomeprazole and lansoprazole. The discontinuation rates were low and consistent across the groups, reflecting the good tolerability of both regimens. Serious adverse events were rare, reinforcing the safety of these therapies.

This study had several limitations that should be addressed in future research. Heterogeneity in study design, particularly the antibiotic combinations, acid suppressant drugs, and bismuth type may have influenced the comparative effectiveness and safety of the therapies. Limited data for certain regimens, such as keveprazan-based and ilaprazole-based BQT, reduce the generalizability of the findings for these treatments. In addition, the lack of long-term follow-up data limits insights into the recurrence rates and resistance patterns. Geographical and population variability, particularly in *H. pylori* antibiotic resistance rates, further complicates the applicability of these results across regions. Moreover, all of the included studies were conducted in East Asian countries and predominantly used the classical BQT (with amoxicillin and clarithromycin) rather than the optimized BQT (tetracycline and metronidazole) as recommended by the American College of Gastroenterology guidelines and the Maastricht VI/Florence consensus report. This limits the generalizability of our findings to populations in other regions.^16^^,^^52^ Future studies should use a standardized protocol or a less-known protocol based on local/regional resistance patterns. Future studies should also elaborate on the long-term effects of P-CAB-based and PPI-based BQT (recurrence rate and resistance pattern) in different patient profiles and regions.

## Conclusion

Pairwise meta-analysis showed that P-CAB-based BQT was slightly more effective than PPI-based BQT in eradicating *H. pylori*, with minimal heterogeneity. However, the NMA showed that vonoprazan-based regimens did not show statistically significant superiority over esomeprazole-based regimens. Both regimens were well-tolerated, with low rrates of adverse events and treatment discontinuation. Based on these findings, vonoprazan- and esomeprazole-based BQT can be recommended as first-line *H. pylori* eradication therapies because of their demonstrated efficacy and favorable safety profiles with considerations for local antibiotic resistance patterns to guide optimal therapy selection. Vonoprazan-based BQT with rapid and consistent acid suppression is particularly suited for patients in regions with high antibiotic resistance or those requiring faster symptom relief. However, esomeprazole-based regimens offer a reliable and widely accessible option, with proven effectiveness in diverse clinical settings. Despite promising results, limitations such as heterogeneity in study design and lack of long-term data highlight the need for standardized protocols and future research.

## Figures and Tables

**Figure S1:**
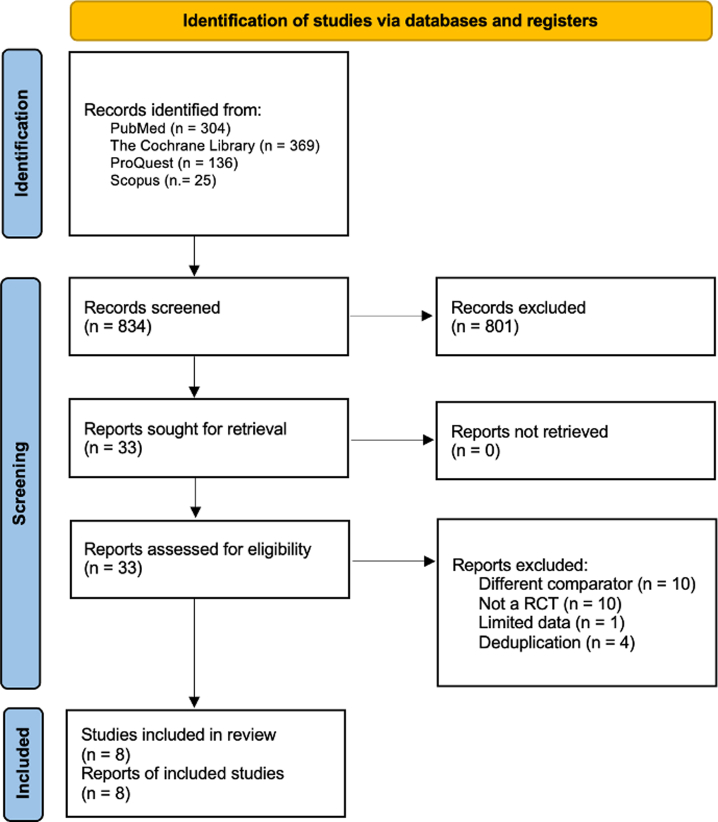


**Figure S2:**
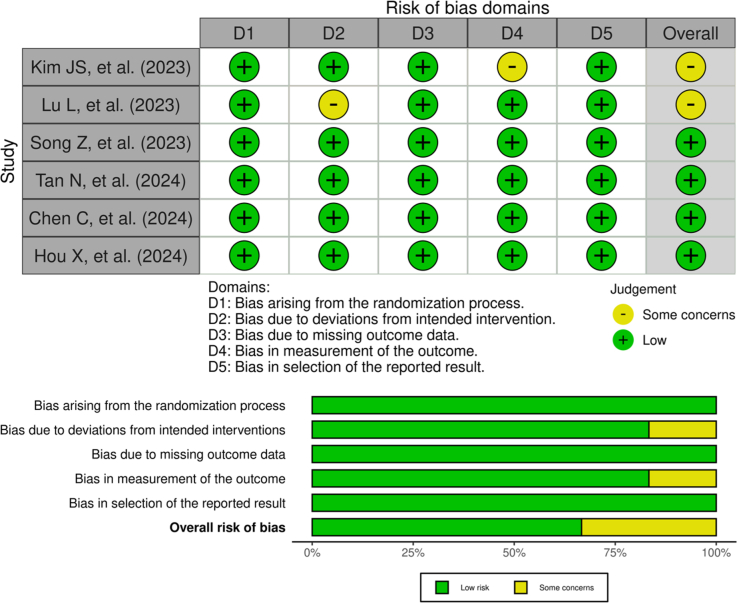

