# Supplementary figures and images for: Comparative Efficacy and Safety of Potassium-Competitive Acid Blocker– and Proton Pump Inhibitor–Based Bismuth Quadruple Therapy for Helicobacter pylori Eradication: A Network Meta-Analysis
Source: Gastro Hep Adv. 2025 May 16;4(9):100705. doi: 10.1016/j.gastha.2025.100705 (PMC12320159; doi:10.1016/j.gastha.2025.100705)

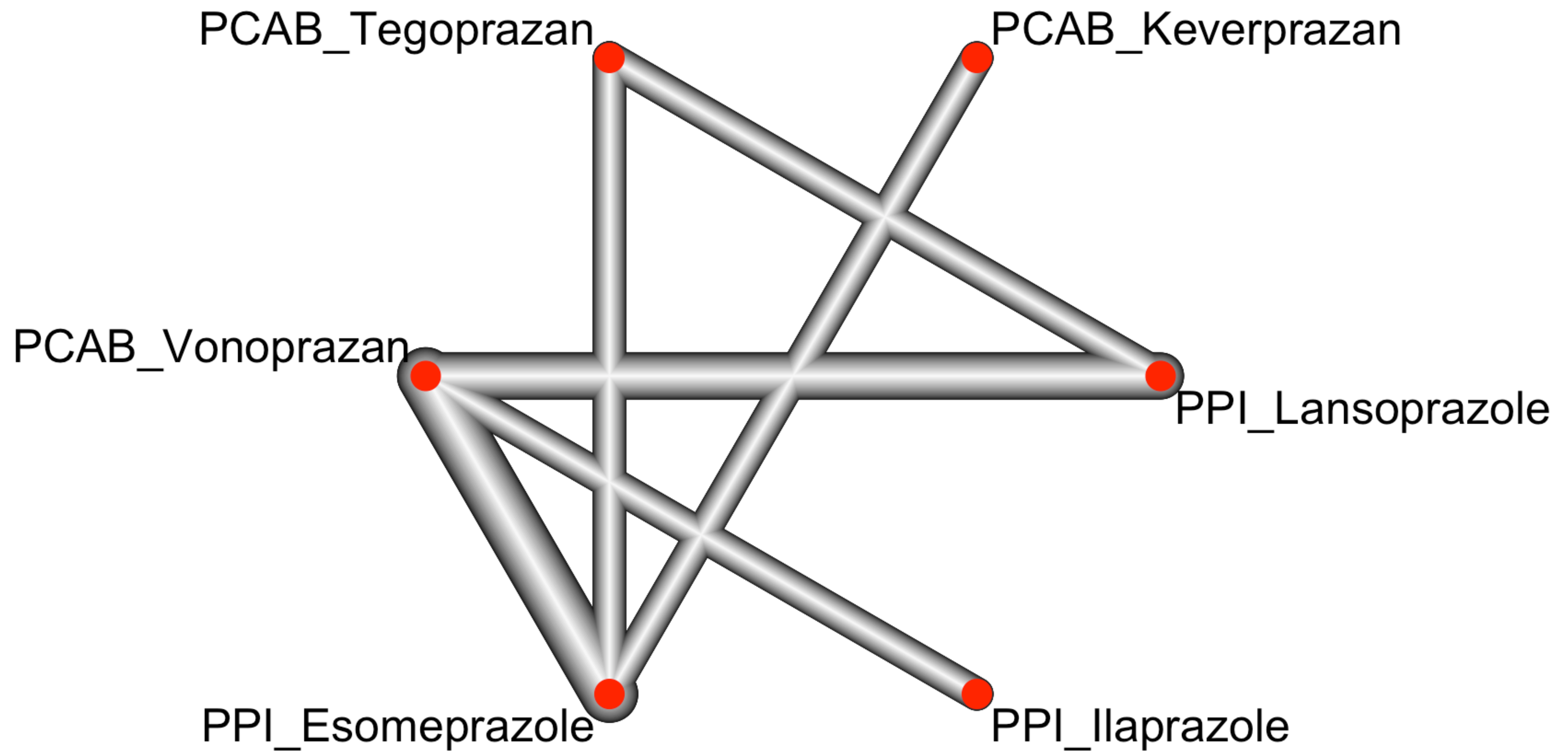

Supplement: Figure S3 [file mmc1.pdf]
